# *Candida* spp. in Cetaceans: Neglected Emerging Challenges in Marine Ecosystems

**DOI:** 10.3390/microorganisms12061128

**Published:** 2024-05-31

**Authors:** Victor Garcia-Bustos, Inmaculada Rosario Medina, Marta Dafne Cabañero Navalón, Alba Cecilia Ruiz Gaitán, Javier Pemán, Begoña Acosta-Hernández

**Affiliations:** 1 Instituto Universitario de Sanidad Animal y Seguridad Alimentaria (IUSA), Universidad de Las Palmas de Gran Canaria, 35001 Arucas, Spain; inmaculada.rosario@ulpgc.es; 2 Severe Infection Research Group, Instituto de Investigación Sanitaria La Fe, 46026 Valencia, Spain; marta.dafne.cabanyero@gmail.com (M.D.C.N.); albacruiz@gmail.com (A.C.R.G.); peman_jav@gva.es (J.P.)

**Keywords:** *Candida*, cetacean candidiasis, marine mammal health, antifungal resistance, zoonotic diseases, ecosystem indicators, emerging fungal pathogens, *Candida* infection

## Abstract

Cetaceans, which are crucial in marine ecosystems, act as sentinels for ecosystem and human–environmental health. However, emerging fungal infections, particularly by *Candida* spp., pose a growing concern in these marine mammals. This review consolidates current knowledge on the prevalence, clinical manifestations, species distribution, and antifungal resistance of *Candida* infections in cetaceans. We detail the diverse pathogenic impacts of *Candida*, including respiratory, dermal, and systemic afflictions, underscoring diagnostic and treatment challenges amid rising antifungal resistance. Our analysis extends beyond health concerns in captive cetaceans, where confinement stress heightens vulnerability, to encompass substantial ecological risks in wild populations. The review emphasizes the One Health perspective, linking cetacean health with broader environmental and human public health issues. We particularly focus on the potential zoonotic transmission of emerging fungal pathogens such as *Candida auris* and the role of environmental changes in fostering antifungal resistance. The study underscores the need for concerted, interdisciplinary efforts in veterinary, medical, and environmental sciences to enhance understanding and management of *Candida* infections in cetaceans. We advocate for comprehensive monitoring and collaborative research initiatives to mitigate the rising challenge of these infections. Addressing *Candida* spp. in cetaceans is not just a conservation priority but a critical step in safeguarding overall marine health and, by extension, human health in the context of evolving infectious diseases.

## 1. Introduction

Cetaceans are a group of marine mammals that include whales, dolphins, and porpoises. They are pivotal in marine ecosystems, affecting predator–prey dynamics and nutrient cycling due to their trophic-level positions [1,2]. They also serve as sentinel species, reflecting ecosystem and human health, attributed to their long lifespans, coastal habitation, high-trophic feeding, and toxin-accumulating fat reserves [3]. As they cohabit in some coastal regions with humans and share dietary sources, cetaceans also highlight human health threats. Their interactions with environmental factors, such as pollution, climate variations, and pathogens, underscore their ecosystem significance [4].

Wild and captive populations are prone to bacterial, viral, fungal, and parasitic infections. Among them, cetacean morbilliviruses, papillomaviruses, *Brucella* spp., and *Toxoplasma gondii*, among others, may disrupt population dynamics through mortality, reduced reproduction, or enhanced virulence of other diseases. Furthermore, the zoonotic risks of marine mammal brucellosis and toxoplasmosis, often misdiagnosed or underreported, especially in developing regions, pose concerns due to unprotected carcass handling and cetacean product consumption [4]. Nevertheless, while most research efforts in marine mammal science focus on bacterial and viral diseases, these infections have long been overlooked, despite sharing many common fungal pathogens such as *Candida* spp. with humans.

Fungal infections in cetaceans pose notable health and conservation challenges. Several fungi, including *Candida*, *Aspergillus*, and *Coccidioides*, have been associated with infections in these marine mammals [5,6,7,8]. *Candida* species, part of the natural microbiota of many hosts, can transition from a commensal to a pathogenic state, causing respiratory, skin, and systemic infections in cetaceans [8,9,10,11,12]. However, there is limited knowledge regarding fungal-associated diseases, and especially *Candida*-associated diseases, in both captive and wild cetaceans.

The objective of this review is to provide a comprehensive understanding of *Candida* spp. infection and colonization in cetaceans, specifically focusing on prevalence, clinical manifestations, species distribution, and antifungal resistance. We aim to summarize the literature on captive and free-ranging cetaceans, highlighting the challenges in accurate diagnosis and potential contributing factors. Additionally, we discuss the emergence of antifungal resistance and identify gaps in knowledge for future research.

## 2. The Emerging Role of *Candida* and Other Fungal Species in Cetacean Health

Fungal diseases pose significant health concerns in cetaceans, with potentially fatal outcomes and challenges in animal care and biodiversity conservation. Among the fungi causing disease in cetaceans, several genera and species have been described. On the one hand, *Candida* species, as well as other fungal pathogens such as *Aspergillus* [5,6,13] and *Coccidioides* [7], have garnered attention due to their involvement in pulmonary, disseminated, and cutaneous fungal infections [8]. On the other hand, *Paracoccidioides ceti* (formerly *Lacazia loboi* or *Loboa loboi*) is the most well-known causative agent of lobomycosis in cetaceans, traditionally considered a zoonotic infection and recognized in human medicine [14,15]. Additionally, various other fungal species have been associated with disease in cetaceans, including *Conidiobolus* [16], *Histoplasma capsulatum* [17], *Cryptococcus gattii* [7,18], *Trichosporon asteroides* [19], *Fusarium* spp. [20,21], *Blastomyces* [22], *Scedosporium angiospermum*, *Parengyodontium album* [23], *Sporothrix schenkii* [24], zygomycetes such as *Rhizopus arrhizus* [25], *Cunninghamella bertholletiae* [26,27], and *Saksenaea vasiformis* or *Apophysomyces elegans* [28].

*Candida* spp. play a significant role in both human and animal health. These infections have remarkable public health concerns due to their impact on vulnerable populations, healthcare-associated infections in humans, rising antifungal resistance in human, veterinary, and environmental health, and high morbimortality of invasive disease in susceptible individuals [9,29,30,31]. *Candida* spp. are part of the microbiota of mucous membranes and are usual colonizers of human and animal hosts [8,32]. In the pathogeny of *Candida* spp. associated infections, several risk factors are combined, such as host susceptibility, disruption of the normal microbiota, adhesion of *Candida* to host tissues, evasion of host immune responses, morphogenetic plasticity and expression of pathogenicity determinants, and the ability to undergo a transition from a commensal to a pathogenic state, ultimately resulting in tissue invasion and infection [9,10,33].

In cetaceans, *Candida* species hold substantial importance, as they have been associated with a range of clinical manifestations and diseases. These infections, particularly those caused by *Candida albicans* and *Candida parapsilosis,* among others, can result in serious health implications, including respiratory tract infections, skin lesions, and systemic fungal dissemination [11,12,34,35,36,37,38,39,40,41,42,43,44,45,46,47]. Its presence also indicates the potential for opportunistic infections, especially in individuals with immunosuppression or those subjected to stressful conditions such as captivity or environmental changes [4,48]. Furthermore, animal sources and, especially, marine mammals have recently been reported to have higher rates of antifungal resistance, a global growing threat [42]. Cetaceans held in captivity are particularly susceptible to these infections due to the stressors associated with confinement and compromised immune systems [10,13,38].

There is a lack of comprehensive knowledge regarding fungal pathogens in the microbiota of both captive and wild cetaceans as well as fungal-associated diseases in these populations. These marine mammals are not easily accessible for research purposes, which results in limited sample sizes and generalizability of the observations. Furthermore, some *Candida* spp. are difficult to identify, and molecular techniques to overcome the species identification limitations of conventional biochemical methods are not widely available or consistently used across studies [49]. The lack of standardized diagnostic criteria for fungal infections and the difficulty of establishing whether *Candida* is the primary cause of an infection, a contaminant in poorly conserved stranded animals, or just a colonizer, add complexity to interpreting results. Hence, the true prevalence and impact of *Candida* infections in cetaceans remain uncertain.

While fungal diseases have been more commonly reported in captive animals, they also have important ecological consequences for wild cetaceans [4]. These diseases have traditionally been underestimated in humans, ecosystems, and animal medicine [50]. Nonetheless, certain fungal diseases can disrupt population dynamics by causing substantial declines in cetacean numbers, especially when coupled with other epizootics, such as morbillivirus outbreaks [4]. The rapid spread of fungal infections through populations could trigger cascading effects on trophic interactions and ecosystem stability.

Cetaceans also serve as valuable ecological indicators of marine ecosystem health. *Candida* spp. and other fungal infections in wild populations can reflect environmental changes, pollution levels, and overall ecosystem disturbances [4,6,47,51,52]. Consequently, delving into their epidemiology or examining the mycobiota in cetaceans can yield valuable insights into ecosystem dynamics while aiding in the assessment of the impacts of anthropogenic activities on marine environments. This becomes particularly relevant considering the emerging challenges posed by the rise of new, highly pathogenic, and resistant fungal species, such as *C. auris*, and the potential emergence of antifungal resistance due to environmental drug pollution and the indiscriminate use of antifungals in agriculture and livestock [53].

The limited understanding of fungal microbiota and fungal infections in wild cetaceans presents a knowledge gap. It is imperative to conduct comprehensive monitoring studies to identify, diagnose, and effectively treat outbreaks or epizootics attributable to these pathogens. By doing so, we can develop robust conservation measures and tackle this challenge head-on.

Fungal diseases in wild cetaceans hold significant implications within the framework of the One Health concept, which acknowledges the interconnectedness of human, animal, and environmental health [54]. The current taxonomic classification system has identified approximately 148,000 fungal species, but it is estimated that the actual number of fungal species could reach up to 5 million, as indicated by high-throughput sequencing methods [55]. However, only a limited subset of these fungal species is recognized as significant pathogens in humans and endothermal animals [56]. Importantly, cetaceans and humans share many of these fungal etiologic agents of infections, indicating potential zoonotic transmission pathways, especially in captive animals [8,15,17,18].

This relatively low incidence of invasive fungal diseases in mammals from a large pool of existing species, including cetaceans and humans, can be attributed to the presence of a complex immune system characterized by adaptive immunity and the physiological trait of endothermy. These factors collectively create a thermal restriction barrier that effectively prevents fungal infections. Only a small number of fungal pathogens have successfully adapted to survive and thrive under high temperatures, enabling them to bypass this barrier and successfully colonize or infect endothermic vertebrates [57,58]. Recent experimental studies have indicated that ongoing climate change and the subsequent rise in environmental temperatures could exert selective pressure, favoring the emergence of thermally tolerant fungal species such as the severe international public health concern of *C. auris* [59].

Certain fungal pathogens that affect cetaceans, including *Candida* spp. can also infect humans, raising concerns regarding potential zoonotic transmission. In this context, *C. auris* has also been hypothesized to have originated from marine environments, and it is yet to be determined if zoonotic behavior has been implicated in the worldwide dissemination of its ancestors [53,59].

Cetaceans could be implicated in the emergence, transmission, and dispersion not only of *Candida* spp. but also other emerging fungal pathogens in the era of global warming. Therefore, a comprehensive understanding of the ecology of these pathogens in cetaceans through a collaborative and interdisciplinary One Health perspective that incorporates veterinary, medical, and environmental expertise is crucial. It would aid in the development of preventive measures that can safeguard cetacean populations and human and environmental health. Comprehensive monitoring studies are needed to identify, diagnose, and effectively treat outbreaks or epizootics attributable to these pathogens. By doing so, we can develop robust conservation measures and tackle this challenge head-on. By gaining insights into the ecology and transmission dynamics of the mycobiota and fungal pathogens in cetaceans, we can better inform strategies for disease prevention and control, benefiting both animal conservation efforts and human well-being.

## 3. Cross-Species *Candida* Infections in Cetaceans: An Evolving Health Concern

*C. albicans* was previously thought to occur in captive cetaceans but not in wild populations [38]. Several cases of *Candida* spp. associated diseases have been previously reported in cetaceans since the 1980s. Symptoms of *Candida* infection in cetaceans often include gastric or esophageal distress, chronic cutaneous lesions (especially by *C. albicans*), respiratory infection [11], and other unspecific symptoms, such as anorexia and reluctance to swallow mouthed food, vomiting, head shaking, and retching. In Germany, *Candida* spp. was isolated from skin lesions of captive freshwater dolphins (*Inia geoffrenis*). In the USA, it was found in the terminal esophagus of Atlantic bottlenose dolphins [24], and *C. albicans* was identified as the etiologic agent of chronic cutaneous candidiasis in captive performing bottlenose dolphins [34]. Furthermore, visceral systemic candidiasis lesions were reported in two deceased killer whales (*Orcinus orca*) in multiple organs, including the heart, kidneys, and lymph nodes [24], but no species identification was carried out. *C. albicans* infection has also been described in an Atlantic bottlenose dolphin, a beluga whale (*Delphinapterus leucas*), a juvenile harbor porpoise (*Phocoena phocoena*), and a pilot whale (*Globicephala melas)* [36] (Table 1).

Systemic candidiasis with unspecific general symptoms such as lethargy and erratic behavior associated with cough, digestive symptoms, cutaneous lesions, and oral thrush with oral ulcers was described in two captive dusky dolphins (*Lagenorrhynchus obscurus*) in South Africa, and isolates of *C. albicans, C. tropicalis,* and *C. parapsilosis* were detected in blowholes, mouth and tongue, feces, and gastric content [37]. Isolates of *Meyerozyma guilliermondii* (formerly *Candida guilliermondii*) and *C. lambica* were found in the blowholes of captive beluga whales, while *C. ciferrii, M. guilliermondii*, and *Kluyveromyces marxianus* (formerly called *C. pseudotropicalis*) were isolated from anal swabs of the same belugas. Other yeasts, including *Debaryomyces hansenii* (formerly called *C. famata* or *Torulopsis candida*)*,* and dimorphic fungi, such as *Trichosporon cutaneum*, have also been isolated from the blowhole and anal swabs of captive beluga whales [39]. However, these early reports sometimes did not reach species sensitivity or provide data on antifungal susceptibility (Table 1).

Recently, there have been increasing reports of various conditions associated with *Candida* species across different taxa, mainly as isolated case reports or case series. *C. albicans* caused bilateral keratomycosis after possible corneal trauma in a captive bottlenose dolphin [45], which was susceptible to both fluconazole and itraconazole. The azole intrinsically resistant species *Pichia kudriavzevii,* formerly *Candida krusei,* was also reported as the cause of chronic intestinal candidiasis resulting in intestinal torsion and death in an aged captive Pacific white-sided dolphin [42]. *Nakaseomyces glabrata* (formerly *Candida* glabrata) was the etiologic agent of a chronic cervical abscess in a US Navy bottlenose dolphin that needed to undergo a surgical approach after being refractory to drainages and repeated antifungal treatment courses [46]. Additionally, in the interesting poster by Mateucci et al. (2022), *Candida* spp. were isolated from the blowhole, gastric fluid, and/or feces in 30 out of 42 samples from 7 captive bottlenose dolphins (2 with mild unspecific symptoms and 1 with intestinal candidiasis) in Italy in 2022. The isolated *Candida* species were identified as *C. albicans* (*n* = 21), *C. tropicalis* (*n* = 6), and *N. glabrata* (*n* = 3), and higher fungal loads were observed in symptomatic animals. All isolated species showed resistance to azoles, while 50% of the isolates were resistant to 5-flucytosine and 23.3% to amphotericin B. No resistance to echinocandins was observed. The study also evaluated the in vitro synergy of three antifungal combinations, fluconazole and terbinafine, voriconazole and terbinafine, and voriconazole and nystatin, and found that the combination of fluconazole and terbinafine exhibited a synergistic effect on 71.4% of tested isolates [12].

Remarkably, *Candida* spp. isolated from cetaceans demonstrate a significantly higher prevalence of antifungal resistance compared to human *Candida* isolates in global clinical settings. This highlights the upcoming public health issue of pharmaceutical pollution in seawater [47]. Schmid and colleagues have previously associated a higher prevalence of azole antifungal resistance with increased halotolerance [40]. However, this must be further validated, and many other factors must be taken into account. In fact, the indiscriminate use of antifungals in large-scale agribusiness and livestock farming and improper disposal of antifungal drugs in wastewater treatment plants and water systems has led to the development of resistance in aquatic yeasts [47]. Moreover, in the context of global warming and global change, the pathogenicity of various *Candida* species has been linked to increasingly severe environments, potentially contributing to the emergence of highly resistant and virulent fungal species such as the superbug *C. auris* [53]. Although information regarding antifungal resistance in wild animals is scarce due to limited access to wild populations, this trend has also been observed in stranded animals such as *C. tropicalis* in dwarf sperm whales (*Kogia sima*) with no previous human contact [42].

Although the evidence is limited, other *Candida* species have also been detected in ill or deceased wild cetaceans or animals from managed populations housed in open ocean enclosures. The last is the case of a respiratory coinfection between *N. glabrata* and parainfluenza virus in a bottlenose dolphin in the USA [43]. Additionally, a coinfection between *Cryptococcus neoformans,* specifically in the teleomorphic state, *Filobasidiella neoformans*, and *C. zeylanoides* was observed in a beached Southern right whale (*Eubalaena australis*) neonate in South Africa [44] (Table 1). However, no studies on the antifungal susceptibility of these isolates have been performed.

## 4. Unmasking Silent Carriers: Azole-Resistant *Candida* in Captive Dolphins

Since the 1980s, research into the mycobiome of cetaceans in captivity began using conventional culture and biochemical techniques. These first insights documented the presence of human-associated yeasts in the feces and pool waters of healthy captive bottlenose dolphins and a beluga whale in Florida, USA [60]. *C. albicans* was isolated from both feces and water samples and was found in high numbers in the asymptomatic beluga whale, which could act as a carrier in the aquarium. *C. tropicalis* was isolated from the feces of three bottlenose dolphins and represented 63% of the total number of dolphin isolates in the study, followed by *C. parapsilosis* and *N. glabrata.* Other less frequent species, such as *C. pelliculosa var. cylindrica, C. humicola*, and *C. bovina*, were isolated once in the animals. However, no data on antifungal susceptibility were reported (Table 1).

Several years ago, the mycobiome of exhalations in healthy captive dolphins in an aquarium in Okinawa, Japan, was studied in 2006 and 2007. The study included two bottlenose dolphins, seven (six in 2007 because of death) Indo-Pacific bottlenose dolphins (*Tursiops aduncus*), three dolphins of F1 offspring between bottlenose dolphins and Pacific bottlenose dolphins, two Pacific white-sided dolphins (*Lagenorhynchus obliquiens*), six false killer whales (*Pseudorca crassidens*), and one rough-toothed dolphin (*Steno bredanensis*) [8]. Fourteen out of twenty were colonized by *Candida* spp. One false killer whale was first colonized by *C. tropicalis* and later by *C. albicans*, while the rest were colonized by the same organism. The prevalence of *C. albicans, C. tropicalis*, and *N. glabrata* was 40%, 30%, and 5%, respectively. *C. albicans* was present in four Indo-Pacific bottlenose dolphins, one bottlenose dolphin, one rough-toothed dolphin, and one F1 offspring between bottlenose dolphins and Pacific bottlenose dolphins. *N. glabrata* was only present in one Indo-Pacific bottlenose dolphin, and *C. tropicalis* was isolated from three false killer whales, one bottlenose dolphin, and two F1 dolphins.

Environmental water samples and samples from working staff were also tested. The prevalence of *Candida* spp. in dolphins, captive pools, and staff members sampled from the oral cavity was 70%, 90%, and 29%, respectively. Interestingly, isolated pathogenic *Candida* species common to the captive dolphins and environments were *C. albicans* and *C. tropicalis*, and identical genotypes in both *Candida* species based on the combination of MDR1 and ITS rDNA were found in some dolphins, between a dolphin and a staff, among dolphins and environments, and environments. This made the authors suggest the potential spread of fungal strains through interspecific transmission and environmental shedding from colonized individuals.

Furthermore, significantly higher rates of antifungal resistance were observed in the isolates. Twenty percent of *C. albicans* isolates were resistant to 5-flucytosine, but no resistance was observed in *C. tropicalis.* More than 86% of *C. albicans* isolates and 80% of *C. tropicalis* isolates showed resistance or dose-dependent susceptibility to fluconazole, and the resistance rates for itraconazole were 80% for both species. However, no data on echinocandins susceptibility were obtained.

Recently, azole-resistant *Candida* species were also isolated from the blowholes of 7 out of 14 bottlenose dolphins in an aquarium in Nagoya, Japan, in 2019. Two individuals were healthy and asymptomatic, but the rest showed clinical signs of respiratory infection. Three dolphins were colonized by *C. albicans*, three by *N. glabrata*, and one by *C. tropicalis.* Different strains were present in some individuals, but no polymicrobial colonization was reported. One isolate of *C. albicans* and two isolates of *C. tropicalis* were resistant to both itraconazole and voriconazole, and three isolates of *N. glabrata* were resistant to itraconazole. Four of the ten isolates were resistant to each azole despite pre- or non-azole therapy [61].

In a recent study by Shirakata et al. (2022) in an aquarium in Enoshima, Japan, eight *C. albicans* strains were isolated from the expired breath of 8 out of 12 dolphins (one Pacific white-sided dolphin and seven bottlenose dolphins) [62]. Remarkably, all isolates were resistant to fluconazole, itraconazole, and voriconazole but exhibited susceptibility to amphotericin B and micafungin. Only one had a history of previous treatment with amphotericin B combined with itraconazole, and three colonized dolphins had been treated with itraconazole. However, a history of previous azole therapy was observed in four individuals who were colonized by azole-resistant *C. albicans* [62].

All data are summarized in Table 1, with the inherent limitations in identifying the colonizing or infective role of *Candida* species in some works.

## 5. Exploring *Candida* Presence in Wild Cetaceans: Early Discoveries and Current Insights

From 1983 to 1986, 19 subsistence-harvested bowhead whales (*Balaena mysticetus*) taken near Barrow, Alaska, were examined for bacterial and fungal microbiomes in both healthy and lesioned skin by conventional culture techniques [64]. Interestingly, many yeast species were isolated, and no significant differences in their occurrence on lesioned versus normal skin were observed, suggesting that yeasts are likely ubiquitous members of their skin microbiota. Against previous knowledge [37], this study from the 1990s provided novel evidence that *Candida* species could not only be present in wild cetacean populations but also colonize healthy individuals as normal tissue microbiota, akin to human hosts.

In this work, several *Candida* species were subjectively observed in non-lesional samples (*C. intermedia*, *C. stellatoidea*, *C. utilis*, *C. inconspicua*, and *M. guilliermondii*), while *D. hansenii*, *P. kudriavzevii*, *C. parapsilosis*, *Diutina rugosa* (formerly *Candida rugosa*), *C. viswanathii*, and *N. glabrata* seemed to be more frequent in lesional skin. Interestingly, there were no reports of *C. albicans*. Additionally, *Candida* species have also been observed in several niches, such as the skin, blowhole, and anus, in three-stranded Atlantic white-sided dolphins (*Lagenorhynchus acutus*) with bacterial septicaemia in Massachusetts, namely, *C. albicans* and *M. guilliermondii* [63], and the first identifications of *C. haemulonii* were also in dolphin skin [66,67]. Moreover, in the study conducted by Buck et al. (1980), it was found that *C. haemulonii* was the predominant *Candida* species isolated from the environmental pools inhabited by captive dolphins [60]. However, *C. haemulonii* was not isolated from the dolphins themselves. The original investigations focused on isolates obtained from seawater and fish belonging to the Haemulidae family, suggesting that the primary source of this fungus could be associated with the thawed fish diet provided to the studied animals, which likely served as a reservoir for *C. haemulonii*.

Later, Buck et al. (2006) conducted a study to isolate and identify aerobic microorganisms, including *Candida* spp., from free-ranging bottlenose dolphins captured and released in the coastal Gulf of Mexico and Atlantic Ocean waters from 1990 to 2002 [32]. The researchers sampled the anus, blowhole, and feces when available from a total of 245 dolphins and found that *Candida* spp. were present in 34% of the sampled dolphins, with a prevalence at 23 out of 35 sampled sites. Of the total isolates, 28% were identified as *C. albicans*, making it the most frequently isolated species, followed by *C. tropicalis*, *N. glabrata* (14%), *C. parapsilosis* (14%), *P. kudriavzevii* (9%), *M. guilliermondii* (5%), *D. hansenii* (5%), and *Clavispora lusitaniae* (formerly *Candida lusitaniae*) (2%). The prevalence of these species in dolphins was 14.3% for *C. tropicalis*, 7.3% for unidentified *Candida* species, 7.0% for *C. albicans*, 3.7% for *D. hansenii*, 3.0% for *C. lusitaniae*, 1.3% for *M. guilliermondii*, and 1.0% for *C. glabrata*.

In another study, Morris et al. (2011) isolated six species of yeasts from blowholes, feces, and gastric fluid of 180 wild bottlenose dolphins captured from two estuarine locations along the southeastern Atlantic Coast of the United States in 2003–2005 [65]. The most abundant species were *N. glabrata* (11.11% of isolates), budding yeasts (10.56%), and *C. tropicalis* (7.8%). *C. albicans* was only present in two samples from the blowhole and gastric fluids, and *D. hansenii* was the third most frequent yeast, with nine isolates mainly from gastric and fecal samples (5%). In addition, two isolates of *D. rugosa* and three isolates of *Trichosporon beigelii* were obtained. However, no antifungal susceptibility studies were performed in either study.

Morris et al. (2011) classified dolphins into three categories of health status (healthy, possibly diseased, or diseased) based on a veterinary panel’s physical examination and complementary tests. Although the researchers observed differences in isolates obtained from gastric swabs among the health classifications, with a greater frequency of *Candida* spp. in cultures from diseased bottlenose dolphins, the differences were not statistically significant [65]. For further structured and summarized information, see Table 1.

## 6. Conclusions

This review highlights the presence of *Candida* species in both captive and free-ranging cetaceans. These fungi have been identified in various biological samples, including feces, pool waters, exhalations, skin, blowholes, and gastric fluids. The most frequently isolated species in captive cetaceans are *C. albicans, C. tropicalis*, *N. glabrata*, and *C. parapsilosis*, which have been found in healthy individuals as well as those displaying signs of mainly respiratory infections and skin lesions. *Candida* colonization appears to be a normal part of cetacean tissue microbiota, similar to humans.

Antifungal resistance is a significant concern in cetaceans, with high rates of resistance observed against fluconazole and itraconazole among *Candida* isolates. Some isolates also exhibit resistance to other antifungal drugs. The potential for interspecific transmission and environmental shedding of *Candida* strains among cetaceans suggests a possible spread of fungal strains within captive environments. Factors such as seawater composition and pharmaceutical pollution may influence the prevalence of antifungal resistance in cetaceans.

Further research is necessary to understand the extent of antifungal resistance in cetaceans, its implications for their health, and the contributing factors. Monitoring and investigation in this field are crucial, especially considering the emergence of highly resistant and virulent fungal species such as *C. auris*. By advancing our knowledge of *Candida* species in cetaceans, we can improve conservation strategies and develop effective management approaches for mitigating the impact of fungal infections and antifungal resistance on these marine mammals.

## Figures and Tables

**Table 1:** Overview of *Candida* spp. colonization and infection in various cetacean species, isolation origins, and antifungal resistance.

*Candida* Species	Cetacean Species	Colonization or Infection	Isolation Origin	Captivity or Free-Living	Antifungal Resistance	Reference
*C. albicans*	*Delphinapterus leucas* *Globicephala melas* *Lagenorhynchus acutus* *Lagenorhynchus obscurus* *Lagenorrhynchis obliquidens* *Orcinus orca* *Phocoena phocoena* *Pseudorca crassidens* *Steno bredanensis* *Tursiops aduncus* *Tursiops aduncus×T. truncatus hybrids* *Tursiops truncatus*	Colonization and infection	Blowhole Cornea Feces Gastric fluid Heart Kidney Lymph node Nares Oral cavity Skin	Captive, free-living	Azole resistance, resistance to 5-flucytosine, resistance to amphotericin B	[8,11,12,32,34,35,36,37,45,60,61,62,63]
*C. bovina*	*Tursiops truncatus*	Colonization	Feces	Captive	No data	[60]
*C. ciferrii*	*Delphinapterus leucas*	Infection	Anus	Captive	No data	[39]
*C. famata*	*Balaena mysticetus Delphinapterus leucas* *Tursiops truncatus*	Colonization and infection	Anus Blowhole Feces Gastric fluid Skin	Captive, free-living	No data	[32,39,64,65]
*N. glabrata*	*Balaena mysticetus* *Tursiops aduncus* *Tursiops truncatus*	Colonization and infection	Blowhole Cervical abscess Feces Gastric fluid Skin	Captive, free-living	Azole resistance	[8,12,32,39,43,46,60,61,65]
*M. guilliermondii*	*Balaena mysticetus* *Delphinapterus leucas* *Lagenorhynchus acutus* *Tursiops truncatus*	Infection, colonization	Anus Blowhole Feces Skin	Captive, free-living	No data	[32,39,63,64]
*C. haemulonii*	Nonreported dolphin	Colonization	Pool water Skin	Free-living	No data	[60,66,67]
*C. humicola*	*Tursiops truncatus*	Colonization	Feces	Captive	No data	[60]
*C. inconspicua*	*Balaena mysticetus*	Colonization	Skin	Free-living	No data	[64]
*C. intermedia*	*Balaena mysticetus*	Colonization	Skin	Free-living	No data	[64]
*P. kudriavzevii*	*Balaena mysticetus* *Lagenorhynchus obliquidens, Tursiops truncatus*	Colonization, infection	Anus Blowhole Feces Intestine Skin	Captive, free-living	Azole intrinsically resistant	[32,39,41,64]
*C. lambica*	*Delphinapterus leucas*	Infection	Blowhole	Captive	No data	[32,39,41,64]
*C. lusitaniae*	*Tursiops truncatus*	Colonization	Anus Blowhole Feces	Free-living	No data	[32]
*C. parapsilosis*	*Balaena mysticetus* *Lagenorrhynchus obscurus* *Tursiops truncatus*	Colonization and infection	Blowhole Feces Gastric fluid Oral cavity Skin	Captive, free-living	No data	[32,37,60,64]
*C. pelliculosa var. cylindrica*	*Tursiops truncatus*	Colonization	Feces	Captive	No data	[60]
*Kluyveromyces marxianus* (formerly *C. pseudotropicalis*)	*Delphinapterus leucas*	Infection	Anus		No data	[39]
*C. rugosa*	*Balaena mysticetus, Tursiops truncatus*	Colonization and infection	Blowhole Gastric fluid Skin	Free-living	No data	[64,65]
*C. stellatoidea*	*Balaena mysticetus*	Colonization	Skin	Free-living	No data	[64]
*C. tropicalis*	*Kogia sima* *Lagenorrhynchus obscurus* *Pseudorca crassidens* *Tursiops aduncus* × *T. truncatus hybrids* *Tursiops truncatus*	Colonization and infection	Blowhole Feces Gastric fluid Oral cavity Skin	Captive, free-living	Azole resistance	[8,12,32,37,42,60,61,65]
*C. utilis*	*Balaena mysticetus*	Colonization	Skin	Free-living	No data	[64]
*C. viswanathii*	*Balaena mysticetus*	Infection	Skin	Free-living	No data	[64]
*C. zeylanoides*	*Eubalaena australis*	Infection	Skin	Free-living	No data	[40]

## Data Availability

No new data were created for this article.
